# Utilizing Technology-Enabled Intervention to Improve Blood Glucose Self-Management Outcome in Type 2 Diabetic Patients Initiated on Insulin Therapy: A Retrospective Real-World Study

**DOI:** 10.1155/2020/7249782

**Published:** 2020-11-10

**Authors:** Jian Lin, Xia Li, Shan Jiang, Xiao Ma, Yuxin Yang, Zhiguang Zhou

**Affiliations:** ^1^National Clinical Research Center for Metabolic Diseases, Key Laboratory of Diabetes Immunology (Central South University), Ministry of Education, and Department of Metabolism and Endocrinology, The Second Xiangya Hospital of Central South University, Changsha 410011, Hunan, China; ^2^Lilly (Shanghai) Management Co., Ltd, No. 288 Shimen No. 1 Road, Jingan District, Shanghai 200041, China; ^3^Lilly Suzhou Pharmaceutical Co., Ltd, No. 288 Shimen No. 1 Road, Jingan District, Shanghai 200041, China

## Abstract

**Background:**

The aim of this study was to assess the benefits of a mobile-enabled app through Lilly Connected Care Program (LCCP) in achieving blood glucose control and adhering to self-monitoring of blood glucose in patients with type 2 diabetes mellitus (T2DM).

**Methods:**

This retrospective study included T2DM patients who were initiated on insulin therapy (mostly premixed insulin) after failure to respond to oral antidiabetic drugs. Patients were provided with glucometers enabled with synchronous data transmission to healthcare providers and family members. The primary objective was to assess the benefits of LCCP based on changes in fasting blood glucose (FBG) and postprandial glucose (PPG) levels from baseline to 12 weeks. Paired *t*-test was used to assess the change in blood glucose (BG) from baseline to week 12.

**Results:**

In total, 14,085 T2DM patients were recruited. Compared with baseline, significant reductions in FBG and PPG were evident at week 12 (FBG: -0.39 mmol/L; PPG: −0.79 mmol/L; both *P* < 0.001). Furthermore, at week 12, the proportion of patients attaining a target glucose level of FBG <7.0 mmol/L and PPG <10.0 mmol/L was 25.37% and 59.68%, respectively, with a statistically significant increase compared with that at baseline (6.74% and 45.59%, respectively, both *P* < 0.001). The frequent monitoring of patients could gain a higher target achievement of FBG (28.1% vs 24.2%) and PPG (64.4% vs 55.1%) than the occasional monitoring patients. Additionally, the incidence of hypoglycemia gradually decreased and was significantly lower than the baseline level.

**Conclusions:**

In T2DM patients with poor glycemic control, the application of mobile enabled intervention (LCCP) along with insulin significantly reduced the hypoglycemia while improving glycemic control during period of naïve initiating insulin therapy. Additionally, the high frequency of BG self-monitoring was associated with better glycemic control.

## 1. Introduction

Diabetes mellitus is a chronic metabolic disorder that has quadrupled in the past three decades and has emerged as a global pandemic associated with substantial socioeconomic burden [1]. The disease is characterized by dysfunction of the glucoregulatory system leading to abnormally elevated hyperglycemia and dysregulation of carbohydrate, protein, and lipid metabolism [2, 3]. According to the recent estimates by the International Diabetes Federation, approximately 463 million adults were diagnosed with diabetes in 2019, and this is projected to increase to 700 million by 2045. [4]. The epidemic of type 2 diabetes mellitus (T2DM) is rapidly emerging in Asia, with China and India being the top two epicenters of the disease crisis [5].

The 2019 treatment guidelines for diabetes published by the American Diabetes Association (ADA) recommend collaborative, multifactorial therapies for improving the long-term outcomes of the disease [6]. Further, the ADA also recommends all diabetic patients to attend self-management educational programs to enable the knowledge, skills, and ability required for diabetes self-care in order to achieve an optimal glycemic control [6, 7]. According to the ADA, diabetes self-management should be patient-centered and is usually recommended to be given individually or in groups or using technology [6]. However, there is not enough knowledge regarding self-management and treatment adherence in Chinese patients with diabetes, and this has been challenging for general practitioners in China [8]. Self-management of diabetes includes dietary monitoring, being physically active, self-monitoring of blood glucose (BG) (SMBG) levels, treatment compliance, healthy coping skills, and adjustments in mental status [9, 10].

A telemedicine system allows remote medical consultations as well as personalized medical advice including lifestyle-related advice from qualified healthcare professionals (HCPs) [11]. The recent advancement in healthcare technology has reported the utility of various technologies in efficient management of these patients by the physicians [11, 12]. The medical devices and diagnostic tools based on these technologies have also been used to provide BG management, thereby improving the treatment compliance [13]. A randomized controlled trial based on computer-based diabetes management program conducted in China reported improved fasting BG (FBG) and postprandial glucose (PPG) levels as well as improved triglyceride levels in the intervention group (conventional treatment + Internet-based glucose monitoring system) compared with the control group (only conventional treatment) [14].

Mobile phones and user-friendly personal messaging applications (apps) have promoted their use in a wider population. People are more equipped to learn mobile apps than computer apps that require higher proficiency [10]. Several studies have reported the benefits of smartphone apps in the management of diabetes [15, 16].

On the basis of these findings, the Lilly Connected Care Program (LCCP) initiated a diabetes management program in China by collaborating with the biggest mobile application (WeChat) as well as healthcare providing platforms in China. This program was introduced to enable patients as well as physicians achieve treatment goals by integrating effective treatment of T2DM, advanced technology, and patient-centric services. In this program, patient's BG readings can be synchronized automatically to their WeChat accounts for viewing and patients are engaged in diabetes education that includes exercise and dietary guidance on need basis at the same time. Moreover, this artificial intelligence-based technology provides patient support model with individualized system-based reminders, self-management support, physician intervention, and call center support. Additionally, this platform also has a channel that allows HCPs to directly guide patients who were in the occurrence or at risk of hypoglycemia or hyperglycemia thus providing a wholesome package for efficient real-time self-management of T2DM. With these characters, we speculated that LCCP platform could assist the physicians in clinical practice to promote SMBG in T2DM patients, which needed to be verified in clinical practices with a large sample size. Thus, we conducted this study to assess the effectiveness of the LCCP-enabled technology platform in BG self-management in diabetic patients newly initiated on insulin analog (mostly premixed insulin analog) therapy. In addition, the study also assessed the patients' adherence and persistence in SMBG for a period of 3 months (12 weeks) on this connected care management system and further evaluated the role of this system on glycemic control, BG monitoring frequency, and the relationship between monitoring frequency and glycemic control in China.

## 2. Materials and Methods

### 2.1. Study Design and Population

This was a retrospective study that included patients with T2DM enrolled in the LCCP program between April 2017 and September 2018. Eligible patients were encouraged by their physicians to register on the LCCP platform. The demographic information, such as gender, age, type of diabetes, and duration of diabetes, was collected at enrollment. Patients could record their BG levels and view their historical BG recordings on the platform. The BG data at week 1 (baseline), week 4, week 8, and week 12 were collected retrospectively. Each patient was followed up for 12 weeks.

The study included (i) patients with T2DM who were aged ≥18 years and newly received insulin analog therapy (mostly initiated with premixed insulin) following failure to achieve the target BG levels with oral antidiabetic drugs in China, (ii) patients presenting with an FBG of ≥7.0 mmol/L and/or a PPG of ≥11.1 mmol/L at baseline, (iii) patients provided informed consent for enrollment in the LCCP program, and (iv) patients starting BG monitoring within 3 weeks after they received a smart glucometer and presenting with FBG and PPG records on the platform at least once a week at week 1 and week 12.

### 2.2. LCCP Program

The HCPs recommended their target patients to join the program via WeChat. After verifying the demographic details of the patients, they were provided with a BG monitoring kit including a free intelligent BG meter and test strips. Once the patients performed their BG tests using this intelligent meter, the results would be synchronized to the mobile WeChat account of patients, HCPs, and invited family members in a real-time scenario. Patient-related data including BG levels and BG monitoring behaviors were shared with the HCPs via the sharing platform. In addition, physicians were notified in cases of hypoglycemia (BG ≤3.9 mmol/L), including extreme hypoglycemia (BG ≤2.8 mmol/L) and hyperglycemia (FBG ≥10mmo/L or PPG ≥13.9 mmol/L) persisting for 3 consecutive days, thereby assisting HCPs to identify abnormal cases in a timely and efficient manner on need basis. In case of abnormal BG readings, the patients would receive reminders/interventions and corresponding diabetes knowledge courses from the system that include dietary, exercise, lifestyle, medication, prevention of complications, and disease management on continuous basis. An inbuilt feature called “daily challenges” provides information on efficient disease management. Further, the patients were rewarded with credits for BG monitoring, diabetes knowledge quiz, and updated antidiabetic regimens. The patient support system consisting of e-courses offered guidance for BG monitoring, dietary advice, and systematic reminder for reinforcing compliance based on the individual BG levels. The courses in the platform were developed on the basis of standards of medical care for type 2 diabetes in China [17] and the self-care courses were based on the American Association of Diabetes Educators 7 Standard of Care [18].

### 2.3. Follow-Up

On the basis of the 2016 Chinese experts consensus statement on premixed insulin in the clinical practice [19], patients initiating premixed insulin analog (twice a day) were advised to monitor their BG twice a day (fasting and before supper) for 3 days in a week. All patients were followed up for 12 weeks.

### 2.4. Study Outcomes

The primary outcome was to assess the benefits of LCCP based on the changes in average FBG and average PPG levels from baseline to 12 weeks. The secondary outcomes included the proportion of patients achieving the BG control target (4.4 mmol/L < FBG <7 mmol/L and/or PPG <10 mmol/L) at 12 weeks, incidence of hypoglycemia, frequency of SMBG, and the association between BG monitoring behavior and glycemic control.

### 2.5. Ethics and Privacy

The study protocol was approved by the ethics committees according to the International Conference for Harmonisation guidelines for Good Clinical Practice and conformed to the Declaration of Helsinki. All patients received information on the purpose and conduct of this study and provided written informed consent.

### 2.6. Statistical Analysis

Descriptive statistics were used to present the baseline characteristics and BG testing. Normally distributed variables are presented as mean and standard deviation (SD); variables that are not distributed normally are presented as median and interquartile range. A paired *t*-test was used to assess the change in average BG from baseline to week 12. McNemar test was used to assess the compliance rate of BG monitoring at week 12 from baseline. Descriptive statistics were used to describe the incidences of hypoglycemia at week 0, week 4, week 8, and week 12. Chi-squared test was used to test the percentage of patients achieving target FBG and PPG in groups with different frequency of monitoring. Statistical analyses were performed using SAS 9.4 software (SAS Institute, Cary, North Carolina) via SAS Enterprise Guide version 7.1. A *P* value of <0.05 was considered as statistically significant.

## 3. Results

### 3.1. Baseline Characteristics

**Figure 1:**
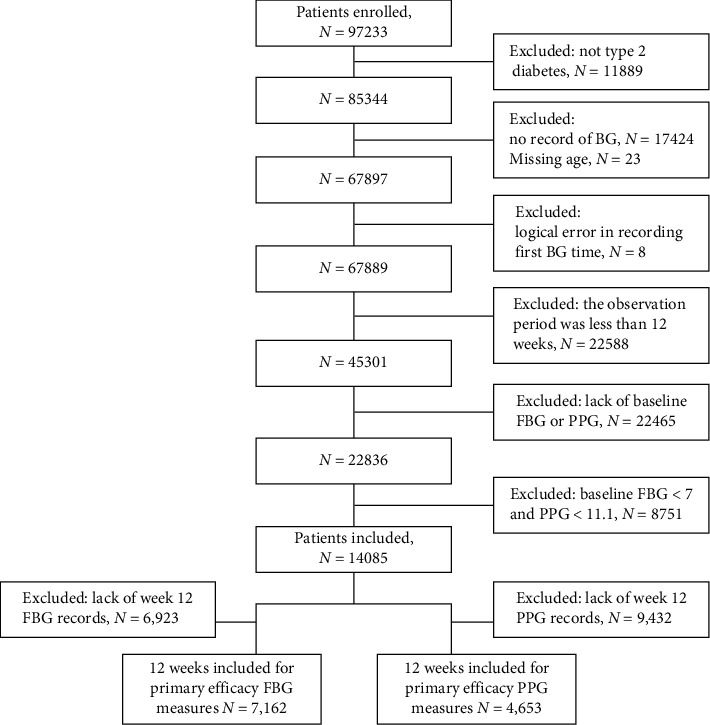
Flow chart of patient inclusion.

A total of 14,085 patients with T2DM were identified in the platform based on the study design and were included in the study. Patient's inclusion flow diagram is shown in Figure 1. The overall baseline characteristics of the patients are presented in Table 1. Among the 14,085 patients, 54.8% were males, with a mean (±SD) age of 51.89 ± 11.96 years. The mean duration of T2DM was 5.47 ± 6.96 years. Before the initiation of insulin, the mean (±SD) FBG and PPG levels were 8.89 ± 2.12 mmol/L and 10.78 ± 3.35 mmol/L, respectively.

### 3.2. Benefits of Glycemic Control in the LCCP Platform

#### 3.2.1. Change in BG Levels

**Figure 2:**
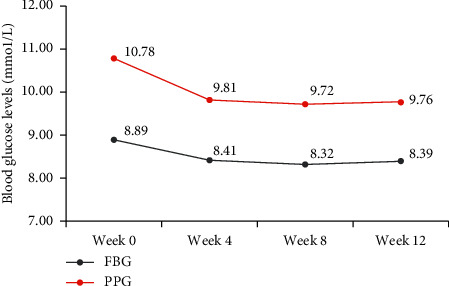
FBG and PPG profiles form baseline to week 12. Mean BG levels are presented. FBG, fasting blood glucose; PPG, postprandial glucose.

Compared with baseline, both FBG and PPG levels at weeks 4, 8, and 12 were significantly reduced (all *P* < 0.001; Figure 2). At week 12, the mean (±SD) FBG and PPG levels were 8.39 ± 2.19 mmol/L and 9.76 ± 3.12 mmol/L, respectively. Compared with baseline, the mean reductions in FBG (mean change: −0.39 mmol/L; 95% CI: −0.45, −0.34) and PPG (mean change: −0.79 mmol/L; 95% CI: −0.68, −0.89) at week 12 are both significant (both *P* < 0.001; Table 2). The mean FBG and PPG levels decreased from baseline to week 12, indicating better overall glycemic control in patients on the LCCP platform.

#### 3.2.2. Proportion of Patients Attaining the Target BG Levels (FBG: 4.4–7 mmol/L; PPG <10 mmol/L)

**Figure 3:**
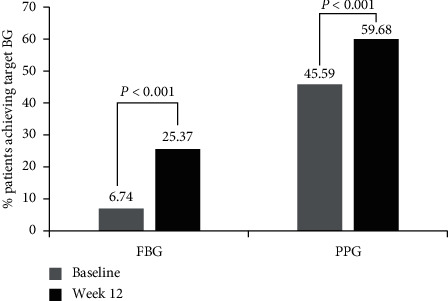
Percentage of patients achieving target FBG and PPG at baseline and week 12. BG, blood glucose; FBG, fasting blood glucose; PPG, postprandial glucose.

Following enrollment on LCCP, there was an increase in the proportion of patients attaining the target FBG and PPG levels. Compared with baseline, a significantly higher percentage of patients at week 12 achieved FBG levels of 4.4 to 7 mmol/L (25.37% versus 6.74%; *P* < 0.001; Figure 3) and PPG levels <10 mmol/L (59.68% versus 45.59%; *P* < 0.001; Figure 3).

### 3.3. Incidence of Hypoglycemia

**Figure 4:**
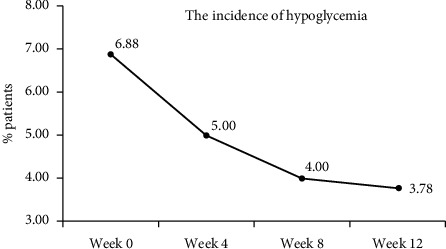
Incidence of hypoglycemia across the time periods. The incidence of hypoglycemic episodes showing the percentage of patients with blood glucose <3.9 mmol/L on the LCCP platform.

The incidence of hypoglycemia decreased gradually in patients on the LCCP platform. The incidences of hypoglycemia at week 4 (5.00%), week 8 (4.00%), and week 12 (3.78%) were all lower than the incidence at baseline (6.88%, Figure 4).

### 3.4. Association between BG Monitoring Frequency and Glycemic Control

**Figure 5:**
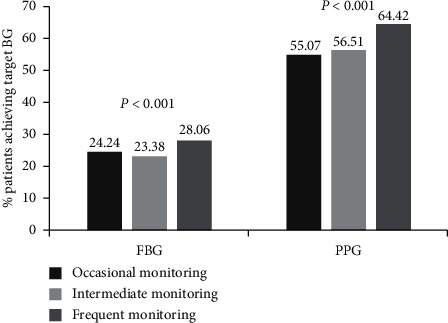
Percentage of patients achieving target FBG and PPG at week 12 with different frequency of monitoring. Occasional monitoring: SMBG <3 times/week; intermediate monitoring: SMBG ≥3 and <6 times/week; frequent monitoring: SMBG ≥6 times/week; BG, blood glucose; FBG, fasting blood glucose; PPG, postprandial glucose; SMBG, self-monitoring blood glucose.

The mean (±SD) SMBG frequency of the patients was observed to be 4.33 ± 3.77 times/week. On the basis of the frequency of BG monitoring, the patients were categorized into frequent monitoring patients, intermediate monitoring patients, and occasional monitoring patients. The frequent monitoring patients were those who performed BG monitoring ≥6 times per week, whereas intermediate monitoring patients performed BG monitoring ≥3 and <6 times per week, and occasional monitoring patients performed BG monitoring <3 times per week. The frequent monitoring patients could gain a higher target achievement of FBG (28.1% versus 24.2%) and PPG (64.4% versus 55.1%; Figure 5) than the occasional monitoring patients. The difference between the groups was statistically significant (*P* < 0.001; Figure 5). These indicate that the high frequency of SMBG is one of important factors contributing to better glycemic control.

## 4. Discussion

We conducted this retrospective study to assess the utility and benefit of the LCCP program in the management of patients with T2DM. In this study involving a large population, the LCCP technology intervention, which was based on smart technology and patient online education, improved the adherence of patients to self-monitoring of their glucose levels, which led to better control of BG levels and hypoglycemic rates.

With a plethora of applications for diabetes management, several advantages of mobile-enabled apps are reported in the literature. A randomized clinical trial on the use of mobile-based apps revealed clinically and statistically significant benefits of an improvement in HbA1c [20]. Furthermore, the use of apps also demonstrated improved physical activity, BG monitoring, and self-management behavior [21, 22].

Evidence from several studies assessing the effects of SMBG by different methods has reported favorable clinical outcomes in terms of glycemic control even in insulin-treated diabetic patients [23–25]. A study by Kato et al. randomly assigned patients to a routine testing group and a structured testing group (group using a chart to record seven‐point BG profile on 3 consecutive days per month). They observed a significant difference in improvement in HbA1c in patients randomized to the structured testing group compared to routine testing group (0.5% decrease versus 0.1% decrease, *P* = 0.002) [23]. Similarly, a study by Kempf et al. reported significantly improved quality of diet and physical activity along with a significant reduction in weight, body mass index, waist circumference, BG, blood pressure, low-density lipoprotein cholesterol, and HbA1c by 0.3% (all *P* < 0.001) in patients following SMBG levels [24]. Further, a study by Kim et al. compared the efficacy of a smartphone-based, patient-centered diabetes care system (mDiabetes) with that of a paper log book (pLogbook) for patients with T2DM. The results demonstrated a significantly greater reduction in HbA1c levels in the mDiabetes group compared with the pLogbook group, and the difference of adjusted mean changes was 0.35% (95% CI: 0.14–0.55; *P* = 0.001) [26]. This present retrospective study showed that after joining the LCCP program, patients with T2DM who had just initiated insulin therapy had significantly lower FBG and PPG levels at 12 weeks compared with baseline. The results of this study were very similar to those reported by Wang et al. wherein the baseline FBG was 7.9 mmol/L, whereas after 3 months of medical treatment and self-monitoring, the mean FBG was 7.0 mmol/L and there was a significant reduction when compared with the control group [27]. Similarly, a study by Sun et al. has reported an improvement in PPG levels after 3 months of mobile phone-based telemedicine application use. In this study, patients in the intervention group (those who were provided with glucometers, capable of data transmission, and received advice pertaining to medication, diet, and exercise via the mHealth telemedicine system) exhibited significantly lower PPG levels in comparison with the control group (those who received routine outpatient care with no additional intervention) (12.09 [3.35] mmol/L vs 13.15 [3.64] mmol/L; *P* = 0.04) [10].

It was also noted that patients in LCCP program attained a higher target glucose level consisting of 4.4 mmol/L < FBG <7 mmol/L and/or PPG at <10 mmol/L compared to baseline. Patients enrolled in the study were continuously assessed and education programs were provided to enhance patients' knowledge of the disease. The improvement in BG levels and the higher proportion of patients achieving the target FBG/PPG compared to baseline may be attributed to the self-monitoring perspective and mindset change, behavior improvement (increase in SMBG monitoring frequency), and self-learning obtained via e-course in the LCCP platform to improve sports and dietary behavior. However, without remote supervision and a support system, it is difficult to achieve sustained efficacy for longer durations of time, and, hence, a long-term follow-up is necessary to assess the overall outcome of any program employed for the management of diabetes mellitus [28].

Previous studies have shown that telemedicine interventions can reduce the rate of hypoglycemia in patients with diabetes. The results of these studies indicate that intensive glycemic control and management by telemedicine may be the plausible reason for reduced hypoglycemic events. Moreover, this approach also reduces the number of face-to-face visits, thereby saving time and resources [29]. In line with the previous studies, in this present study, there was a significant reduction in the rate of hypoglycemia following enrollment in the LCCP program. This might be due to the fact that LCCP platform provides free glucometers, strips, lancets, quick screening and identification of abnormal issues, strong connection between HCP and patients, and personal cares/educational courses/compliance reinforcement. In detail, patients could receive diet and exercise intervention on time which facilitates them to handle hypoglycemia and also to prevent occurrence of hypoglycemia. Meanwhile, with the stability of insulin dose titration and the patient's ability of handling the treatment adjustments, the risk of hypoglycemia reduces. As LCCP platform can capture both documented and symptomatic hypoglycemia and documented asymptomatic hypoglycemia, we believe that we could capture most of the hypoglycemia events by LCCP platform; thus, the decline in the incidence of hypoglycemia after using LCCP reflects the effectiveness of the LCCP platform in monitoring and control of BG which are all features facilitating self-monitoring of daily BG and may further lead to improvement in glucose control.

We also assessed the impact of monitoring frequency on the overall BG control. Patients who followed the instructions appropriately and performed blood monitoring at least 6 times per week reported improved FBG and PPG levels in comparison with those who were irregular in monitoring their BG. The mobile intelligent management integrated solution can help improving the compliance of the SMBG. In general, mobile-based applications facilitate good glycemic control and management in patients with T2DM. This study was based on real-world data with a large sample. However, there are several limitations to the study. First, the study is based on real-world data, and information on diet habits, physical exercise, combined medication, chronic complications, education, and socioeconomic status was not collected. These factors may have an impact on glycemic control and monitoring behavior. Second, the observation period was short. In order to assess the overall outcome of any program employed for the management of diabetes mellitus, a long-term follow-up is necessary. Third, in this study, we did not assess the value of HbA1c, which is the most crucial primary endpoint for BG improvement, as it is an average of blood glucose levels over the preceding weeks or months. Fourth, as it is a real-world study, the rate of patients lost to follow-up is relatively high. Fifth, the lack of proper control is a major drawback. Finally, there is no reported assessment of change in adherence or SMBG monitoring frequency in this study. The potential reasons for the improvement in BG control and monitoring behavior need to be further investigated. However, our results provide an evidence for effectiveness of use of LCCP platform on diabetes management in real-world settings and further randomized controlled trials are warranted to confirm the same.

## 5. Conclusions

The LCCP is a comprehensive disease management program that provides useful tools for T2DM management. Patients enrolling in the mobile-enabled program had significantly improved BG and decreased hypoglycemic incidence compared to baseline. Better glycemic control was observed in patients who monitored their BG more frequently compared to those who “occasionally monitored” their BG. Further, long-term results are required to confirm the effectiveness of the program in achieving and maintaining glycemic control.

## Figures and Tables

**Table 1:** Patient characteristics at baseline.

Characteristics	Total patients
Number of subjects	14,085
Gender	
Male	7,712 (54.8%)
Female	6,373 (45.2%)
Age (years) (continuous)	51.89 ± 11.96
Age (years) (categorical)	
<60	10,287 (73.0%)
≥60	3,798 (27.0%)
Duration of T2DM (years)	5.47 ± 6.96
Region	
Northeast	2,602 (18.5%)
North China	3,104 (22.0%)
East China	3,626 (25.7%)
South China	599 (4.3%)
Central China	2,342 (16.6%)
Southwest	1,167 (8.3%)
Northwest	644 (4.6%)
Missing	1 (0.0%)
FBG (mmol/L) (continuous)	8.89 ± 2.12
FBG (mmol/L) (categorical)	
<7	986 (7.0%)
7–9	8,011 (56.9%)
9–11	3,181 (22.6%)
≥11	1,907 (13.5%)
PPG (mmol/L) (continuous)	10.78 ± 3.35
PPG (mmol/L) (categorical)	
<9	4,490 (31.9%)
9–11	3,682 (26.1%)
11–13	3,002 (21.3%)
≥13	2,911 (20.7%)

Data are presented as mean ± SD or as *n* (%). FBG, fasting blood glucose; PPG, postprandial glucose; T2DM, type 2 diabetes mellitus.

**Table 2:** Change from baseline at week 12 for primary efficacy (FBG and PPG) measures.

	Baseline	Endpoint	Mean change from baseline (95% CI)	*P* value
FBG (mmol/L)	*n* = 14085	*n* = 7162	−0.39 (−0.45, −0.34)	<0.001
	8.89 ± 2.12	8.39 ± 2.19		
PPG (mmol/L)	*n* = 14085	*n* = 4653	−0.79 (−0.68, −0.89)	<0.001
	10.78 ± 3.35	9.76 ± 3.12		

Unless indicated otherwise, data are presented as mean ± SD. CI, confidence interval; FBG, fasting blood glucose; *n*, number of patients; PPG, postprandial glucose.

## Data Availability

The raw data used to support the findings of this study will be provided upon request.
